# Polygenic risk for alcohol dependence associates with alcohol consumption, cognitive function and social deprivation in a population‐based cohort

**DOI:** 10.1111/adb.12245

**Published:** 2015-04-10

**Authors:** Toni‐Kim Clarke, Andrew H. Smith, Joel Gelernter, Henry R. Kranzler, Lindsay A. Farrer, Lynsey S. Hall, Ana M. Fernandez‐Pujals, Donald J. MacIntyre, Blair H. Smith, Lynne J. Hocking, Sandosh Padmanabhan, Caroline Hayward, Pippa A. Thomson, David J. Porteous, Ian J. Deary, Andrew M. McIntosh

**Affiliations:** ^1^ Division of Psychiatry; ^2^ Division of Human GeneticsDepartment of PsychiatryYale University School of MedicineVA CT Healthcare CenterWest HavenCTUSA; ^3^ Medical Scientist Training Program and Interdepartmental Neuroscience ProgramYale University School of MedicineWest HavenCTUSA; ^4^ Department of Genetics and NeurobiologyYale University School of MedicineWest HavenCTUSA; ^5^ Department of PsychiatryUniversity of Pennsylvania Perelman School of MedicineVISN4 MIRECC, Philadelphia VA Medical CenterPhiladelphiaPAUSA; ^6^ Departments of Medicine, Neurology, Ophthalmology, Biomedical Genetics, Epidemiology, and BiostatisticsBoston University School of Medicine and Public HealthBostonMAUSA; ^7^ Population Health SciencesUniversity of DundeeUK; ^8^ Division of Applied Health SciencesUniversity of AberdeenUK; ^9^ Institute of Cardiovascular and Medical SciencesUniversity of GlasgowUK; ^10^ Centre for Genomics and Experimental MedicineInstitute of Genetics and Molecular MedicineWestern General HospitalUniversity of EdinburghUK; ^11^ MRC Human GeneticsMRC IGMMUniversity of EdinburghUK; ^12^ Centre for Cognitive Ageing and Cognitive EpidemiologyUniversity of EdinburghUK; ^13^ Department of PsychologyUniversity of EdinburghUK

**Keywords:** Alcohol dependence, cognition, environment, genetics, polygenic, social deprivation

## Abstract

Alcohol dependence is frequently co‐morbid with cognitive impairment. The relationship between these traits is complex as cognitive dysfunction may arise as a consequence of heavy drinking or exist prior to the onset of dependence. In the present study, we tested the genetic overlap between cognitive abilities and alcohol dependence using polygenic risk scores (PGRS). We created two independent PGRS derived from two recent genome‐wide association studies (GWAS) of alcohol dependence (SAGE GWAS: *n* = 2750; Yale‐Penn GWAS: *n* = 2377) in a population‐based cohort, Generation Scotland: Scottish Family Health Study (GS:SFHS) (*n* = 9863). Data on alcohol consumption and four tests of cognitive function [Mill Hill Vocabulary (MHV), digit symbol coding, phonemic verbal fluency (VF) and logical memory] were available. PGRS for alcohol dependence were negatively associated with two measures of cognitive function: MHV (SAGE: *P* = 0.009, β = −0.027; Yale‐Penn: *P* = 0.001, β = −0.034) and VF (SAGE: *P* = 0.0008, β = −0.036; Yale‐Penn: *P* = 0.00005, β = −0.044). VF remained robustly associated after adjustment for education and social deprivation; however, the association with MHV was substantially attenuated. Shared genetic variants may account for some of the phenotypic association between cognitive ability and alcohol dependence. A significant negative association between PGRS and social deprivation was found (SAGE: *P* = 5.2 × 10^−7^, β = −0.054; Yale‐Penn: *P* = 0.000012, β = −0.047). Individuals living in socially deprived regions were found to carry more alcohol dependence risk alleles which may contribute to the increased prevalence of problem drinking in regions of deprivation. Future work to identify genes which affect both cognitive impairment and alcohol dependence will help elucidate biological processes common to both disorders.

## Introduction

Alcohol dependence is characterized by a maladaptive pattern of alcohol consumption that can lead to tolerance, withdrawal and a loss of control over intake that has negative psychological and physiological consequences. Cognitive impairment is a common feature of alcohol dependence (Hester, Lubman & Yucel 2010) and persists after alcohol detoxification in 50–75 percent of cases (Smith & Atkinson 1995; Parsons & Nixon 1998). The relationship between alcohol dependence and cognitive impairment is complex. Cognitive impairment may increase the risk of alcohol dependence or arise as a consequence of prolonged heavy drinking. Family studies comparing non‐alcoholic children of alcoholics to those with a negative family history of alcoholism find those with alcoholic parents perform worse on tests of executive function (Ozkaragoz, Satz & Noble 1997; Gierski *et al*. 2013) suggesting that cognitive impairment may precede the onset of dependence.

Epidemiological studies have shown higher childhood IQ to be associated with less alcohol‐induced hangovers in adulthood (Batty, Deary & Macintyre 2006). However, higher childhood mental ability has also been associated with increased alcohol intake and alcohol‐related problems in adulthood (Batty *et al*. 2008). The relationship between alcohol dependence and cognitive ability is confounded by environmental exposures that obscure observational associations and causality. Social deprivation and the number of years spent in education correlate strongly with cognitive ability. Alcohol consumption is positively correlated with socio‐economic status and education level (Huerta & Borgonovi 2010; Corley *et al*. 2011; Grittner *et al*. 2012); however, problem drinking is more prevalent in regions of social deprivation (Bromley *et al*. 2012). These factors interact as the effect of socio‐economic status negatively impacts cognitive function in individuals with a positive family history of alcoholism (Lovallo *et al*. 2013). Furthermore, social deprivation and education have a substantial genetic component that has been shown to overlap with the genetic basis of cognitive ability in this sample (Marioni *et al*. 2014).

A common genetic aetiology may explain some of the overlap between alcohol dependence and cognitive impairment. The estimated heritabilities for alcohol dependence and adult general cognitive ability range from ∼40 to 70 percent (Enoch & Goldman 2001; Haworth *et al*. 2010; Calvin *et al*. 2012); however, few studies have examined their genetic overlap. One established approach to detect shared genetic effects between traits is to use polygenic risk profiling (Purcell *et al*. 2009; Evans *et al*. 2013). Summary data from a genome‐wide association study (GWAS) of a disease of interest is used to determine the weighted number of risk alleles an individual in an independent sample carries. Polygenic risk profiles thus denote an individual's genetic load for a particular disorder. By testing the association between a polygenic risk score (PGRS) for alcohol dependence and potential biological intermediates (cognitive ability), we are able to analyse the relationship between the two traits without having to measure alcohol dependence directly in the cohort being studied. Furthermore, associations between polygenic risk profiles and biological intermediates will not be confounded by environmental exposures and may highlight potentially causal pathways which warrant further study (Evans *et al*. 2013).

In the present study, we calculated two independent polygenic risk profiles for alcohol dependence in 9863 members of Generation Scotland: Scottish Family Health Study (GS:SFHS), a population‐based epidemiological cohort (Smith *et al*. 2006). Two PGRS were created using summary data from two independent European‐American GWAS of alcohol dependence (SAGE, *n* = 2750; Yale‐Penn, *n* = 2377) (Gelernter *et al*. 2013). The GWAS summary data were meta‐analysed and a third PGRS created from the meta‐analysis data. The Yale‐Penn and SAGE datasets are the discovery samples from which GWAS summary statistics were derived and GS:SFHS is the sample in which PGRS were created and analysed. Alcohol dependence was not measured in GS:SFHS individuals and therefore polygenic risk profiles were used to explore the genetic relationship between alcohol dependence, cognitive ability, education and social deprivation.

## Methods

### Generation Scotland: Scottish Family Health Study

GS:SFHS is a family‐based epidemiological cohort recruited at random through general medical practices across Scotland. The protocol for recruitment is described in detail elsewhere (Smith *et al*. 2006). The cohort consists of 21 516 individuals over 18 years of age recruited if they had at least one other family member willing to participate. Genome‐wide single nucleotide polymorphism (SNP) data are available for 9863 individuals, and these are the individuals who are described and whose data are used in the present study (mean age = 52.2, SD = 13.64) (5788 female, 4075 male).

Demographic information available included socio‐economic deprivation measured using the Scottish Index of Multiple Deprivation (SIMD) 2009 matched to each participant's postcode (The Scottish Government 2009). SIMD is not a direct measure of an individual's socio‐economic status, but is a ranking for their local area (6505 areas in total with an area population mean of ∼800). It is derived from data on employment, income, health, education, housing, crime and access to services. SIMD is a rank number from 1 to 6505 and the lower the number the more socially deprived the geographical region. Each participant self‐completed a pre‐clinic questionnaire, which included information on their education by asking, ‘how many years altogether did you attend school/study full‐time?’

Cognitive abilities were assessed using four tests. Verbal ability was assessed using the Mill Hill Vocabulary Scale, junior and senior synonyms (Raven 1965). Immediate and delayed scores from the recall section of one story of the Wechsler Logical Memory test were summed to provide a measure of verbal declarative memory (Wechsler 1998). The Wechsler Digit Symbol Coding test was used to measure processing speed (Wechsler 1998). Executive function was measured using the letter‐based phonemic verbal fluency test (letters C, F and L for 1 minute each) (Lezak 1995).

Alcohol consumption was assessed using a pre‐clinical questionnaire. Participants were identified as current drinkers, former drinkers or never drinkers. Consumption was measured in self‐reported units of alcohol consumed in the previous week and converted into grams of alcohol/kg/week by multiplying units by 7.9 and dividing by the participant's weight (measured in the research clinic) in kilograms. All components of GS:SFHS have received ethical approval from the NHS Tayside Committee on Medical Research Ethics (REC Reference No. 05/S1401/89).

### Genotyping and quality control

Blood samples were collected using standard operating procedures and stored at the Wellcome Trust Clinical Research Facility Genetics Core, Edinburgh (www.wtcrf.ed.ac.uk) where they were genotyped using the IlluminaHumanOmniExpressExome ‐8v1.0 BeadChip and Infinum chemistry (Gunderson 2009). The genotypes were then processed using the IlluminaGenomeStudio Analysis software v2011.1. The details of blood collection and DNA extraction are provided elsewhere (Smith *et al*. 2006).

### Polygenic profiling

Genotyping quality control was performed and SNPs with a minor allele frequency < 5 percent, significant deviation from Hardy‐Weinberg equilibrium (*P* < 0.001), or a call rate < 98 percent were removed from further analyses. Individuals with genotyping call rates lower than 95 percent were also removed. Any strand‐ambiguous SNPs were removed from the GS:SFHS dataset and genotypes were LD pruned using clump‐based pruning (*r* ^2^ = 0.25, 300 kb window) to create a set of SNPs in linkage equilibrium. GS:SFHS genetic data included only raw genotypes (unimputed data) and therefore only SNPs common to the Yale‐Penn/SAGE samples and GS:SFHS were used to create polygenic scores.

**Figure 1:**
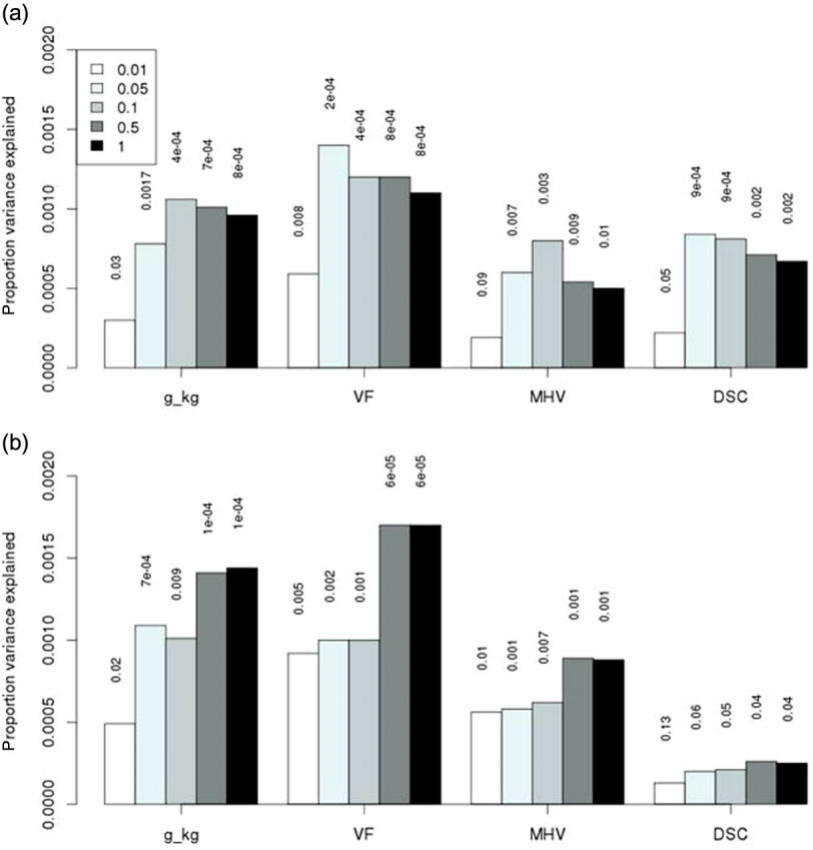
(a) Proportion of variance in alcohol consumption and cognitive variables explained by SAGE AD polygenic risk score derived using five different *P*‐value thresholds. (b) Proportion of variance in alcohol consumption and cognitive variables explained by Yale‐Penn AD polygenic risk score derived using five different *P*‐value thresholds. DSC = digit symbol coding; g_kg = grams of ethanol per kg of body weight consumed per week; MHV = Mill Hill vocabulary; VF = verbal fluency. Only tests significantly associated with AD polygenic risk score from both Yale‐Penn and SAGE GWAS at the *P*‐value threshold *P* ≤ 0.5 are presented.

PGRS for alcohol dependence were created in GS:SFHS using PLINK as previously described in detail (Purcell *et al*. 2009) using the summary GWAS data from the two independent (SAGE and Yale‐Penn) GWAS of alcohol dependence (Gelernter *et al*. 2013). Both the Yale‐Penn and SAGE alcohol dependence GWAS used an ordinal model to test for association. The imputed SNP allele dosage was the dependent variable and DSM‐IV symptom counts for alcohol, cocaine and opioid dependence (adjusted for age, sex and ancestry principal components) were ordinal predictors. For 5 708 204 high‐quality SNPs with *P*‐values in both the Yale‐Penn and SAGE datasets, inverse variance meta‐analysis was performed using METAL (Willer, Li & Abecasis 2010). These summary data were used to create a meta‐analysis PGRS for alcohol dependence (*n* = 5127) in GS:SFHS. PGRS were created using SNPs associated with alcohol dependence with *P*‐value thresholds of 0.01, 0.05, 0.1, 0.5 and 1 in the Yale‐Penn and SAGE GWAS; however, only data from the *P*‐values for the ≤ 0.5 threshold are presented in the tables as these generally explained the largest amount of variance in the dependent variable (Fig. 1 shows significance and variance explained at all *P*‐value thresholds).

A large majority (87 percent) of participants were born in Scotland and 95 percent in the United Kingdom. Roughly 82 percent of parents and 75 percent of grandparents were also born in Scotland. Up to 99 percent of participants defined their ethnicity as ‘white’ (Smith *et al*. 2013). Multi‐dimensional scaling (MDS) components were created according to the ENIGMA 1000 genomes protocol (ENIGMA 2013) in the software package PLINK (Purcell *et al*. 2007). A plot of the first and second MDS components showing the genotyped individuals in GS:SFHS and 11 different ethnic HapMap populations is shown in Supporting Information Fig. S1. The GS:SFHS individuals cluster tightly with the HapMap CEU population and show little admixture with the other ethnic populations. Four MDS components were used to correct for population stratification.

The PGRS presented throughout the manuscript were created using all autosomal SNPs. However, as several known alcohol metabolism genes are located on chromosome 4q23 (*ADH4*, *ADH5*, *ADH6*, *ADH1A*, *ADH1B*, *ADH1C*), a separate PGRS was calculated with chromosome 4 SNPs excluded to provide an estimate of polygenic risk for alcohol dependence that was independent of the SNPs within these loci.

### Statistical analysis

All variables were transformed towards normality where necessary using the Box‐Cox transformation procedure implemented in the MASS package in R (Venables & Ripley 2002). The ASReml‐R (www.vsni.co.uk/software/asreml) software package was used to implement mixed linear model association analyses. Family structure was fitted as a random effect by creating an inverse of a relationship matrix using pedigree kinship information. All variables were scaled to have a mean of 0 and a standard deviation of 1, and the reported values of beta are standardized. Wald's conditional *F*‐test was used to calculate *P*‐values for fixed effects. The proportion of variance explained by PGRS was calculated by taking the change in the sum of the residual variance and the additive genetic variance after removing the PGRS from the model, then dividing this by the sum of residual variance and the additive genetic variance. Pearson's correlations were used to determine the correlation between the two PGRS and alcohol consumption and cognitive/demographic variables in unrelated individuals (*n* = 6413) (Supporting Information Table S1).

The validity of the alcohol dependence PGRS was analysed by testing for association between polygenic risk for alcohol dependence and alcohol consumption. We then tested the association with cognitive ability test scores. Alcohol consumption was included as a fixed‐effect covariate for all analyses except where alcohol consumption was the dependent variable. Any association between polygenic risk for alcohol dependence and cognitive ability would therefore reflect genetic overlap between the traits without the confounding effect of alcohol consumption. Two models were fit to test these associations: an unadjusted model which had age, sex, alcohol consumption, four MDS components and the PGRS as fixed effects. An adjusted model was also fitted which included social deprivation and education as additional fixed effects.

To test the interaction of SIMD and PGRS with cognitive measures as the dependent variable, age, sex, alcohol consumption, PGRS and SIMD were included as main effects and the interaction term ‘polygenic risk score*SIMD’. To control for confounders, each covariate was entered as an interaction with the genetic (PGRS) and environmental (SIMD) effect as recommended elsewhere (Keller 2013). The *P*‐values presented are raw *P*‐values uncorrected for multiple testing. False discovery rate (FDR) was implemented in the R package ‘fdrtools’ to estimate the local FDR.

## Results

### Relationship of polygenic risk for alcohol dependence to alcohol consumption

The Pearson's correlation between the two PGRS for alcohol dependence derived from the SAGE and Yale‐Penn GWAS was 0.61. Polygenic risk for alcohol dependence in GS:SFHS was positively associated with alcohol consumption (SAGE: β = 0.045, *P* = 0.00003; Yale‐Penn: β = 0.042, *P* = 0.00009) (Table 1). The proportion of variance in alcohol consumption explained by risk scores was low: less than 0.2 percent (Fig. 1). Using the PGRS derived from the meta‐analysis of the SAGE and Yale‐Penn GWAS, the effect size and variance explained increased modestly (β = 0.049, *P* = 2.8 × 10^−6^, *r* ^2^ = 0.0022) (Table 1). The SAGE and Yale‐Penn alcohol scores remained significantly associated with alcohol consumption when chromosome 4 SNPs were removed (Yale‐Penn: β = 0.034, *P* = 0.001, *r* ^2^ = 0.00102 and SAGE: β = 0.036, *P* = 0.0006, *r* ^2^ = 0.0012) (Supporting Information Table S1). The effect size (β) was reduced by 14–24 percent (Yale‐Penn and SAGE scores, respectively). Although chromosome 4 SNPs explain some of the variance in alcohol consumption, a significant polygenic risk signal is present on the remaining chromosomes.

**Table 1:** Association of AD polygenic risk with alcohol consumption, education and SIMD. All associations are adjusted for age, sex and four MDS components to adjust for population stratification. Covariate adjusted models are presented with the inclusion of the other traits as covariates, e.g. alcohol consumption is adjusted for education and SIMD

Trait	SAGE AD polygenic risk score		SAGE AD polygenic risk score + covariates		SAGE AD polygenic risk score		SAGE AD polygenic risk score + covariates		Meta‐analysis AD polygenic risk score		Meta‐analysis AD polygenic risk score + covariates	
	Stats	*P*	Stats	*P*	Stats	*P*	Stats	*P*	Stats	*P*	Stats	*P*
Alcohol consumption	β = 0.035, *r* ^2^ = 0.001	**0.0007**	β = 0.045, *r* ^2^ = 0.0019	**0.00003**	β = 0.04, *r* ^2^ = 0.0014	**0.00012**	β = 0.042, *r* ^2^ = 0.0017	**0.00009**	β = 0.049, *r* ^2^ = 0.002	**2.8 × 10^−6^**	β = 0.056, *r* ^2^ = 0.003	**2.1 × 10^−7^**
Education	β = −0.055, *r* ^2^ = 0.0028	**2.6 × 10^−7^**	β = −0.043, *r* ^2^ = 0.0016	**0.00006**	β = −0.033, *r* ^2^ = 0.0009	**0.0021**	β = −0.022, *r* ^2^ = 0.0003	0.035	β = −0.049, *r* ^2^ = 0.0021	**5.4 × 10^−6^**	β = 0.035, *r* ^2^ = 0.001	**0.001**
SIMD	β = −0.054, *r* ^2^ = 0.0028	**5.2 × 10^−7^**	β = −0.044, *r* ^2^ = 0.0018	**0.00005**	β = −0.047, *r* ^2^ = 0.002	**0.000012**	β = −0.046, *r* ^2^ = 0.0018	**0.00003**	β = −0.063, *r* ^2^ = 0.004	**1.1 × 10^−8^**	β = 0.056, *r* ^2^ = 0.003	**2.6 × 10^−7^**

*P*‐values highlighted in bold remain significant after FDR correction for multiple comparisons. Polygenic risk scores derived from SNPs with a GWAS *P*‐value ≤ 0.5 are presented.

### Relationship of polygenic risk for alcohol dependence to socio‐economic status and education

Social deprivation and the number of years in education were negatively associated with polygenic risk for alcohol dependence in GS:SFHS. Individuals living in more socially deprived regions (SAGE: β = −0.044, *P* = 0.00005 & Yale‐Penn: β = −0.046, *P* = 0.00003) and who had spent fewer years in education (SAGE: β = −0.043, *P* = 0.00006; Yale‐Penn: β = −0.022, *P* = 0.04) had a significantly higher polygenic risk for alcohol dependence even after covariate adjustment (Table 1). As Mill Hill Vocabulary test performance is correlated with social deprivation and education (*r* = 0.24, *P* =< 2.2 × 10^−16^) (Supporting Information Table S2), the association between alcohol dependence polygenic risk and deprivation was also tested in GS:SFHS after controlling for Mill Hill Vocabulary. The negative association between polygenic risk and SIMD has a similar effect size after controlling for Mill Hill Vocabulary (SAGE: *P* = 0.000007, β = −0.047; Yale‐Penn: *P* = 0.0002, β = −0.039). However, education was not significantly associated with polygenic risk, and the effect sizes were markedly reduced, after the same adjustment (SAGE: *P* = 0.44, β = −0.008; Yale‐Penn: *P* = 0.41, β = 0.008). The amount of variance in education and SIMD explained by polygenic risk for alcohol dependence was less than 0.36 percent across all models. The association between the meta‐analysis PGRS and demographic and cognitive variables is presented in Tables 1 and 2.

**Table 2:** Relationship between SAGE, Yale‐Penn and meta‐analysis polygenic risk score and cognitive variables

Polygenic risk score	Verbal fluency			Mill Hill Vocabulary			Digit symbol coding			Logical memory		
	Beta (SE)	*r* ^2^	*P*‐value	Beta (SE)	*r* ^2^	*P*‐value	Beta (SE)	*r* ^2^	*P*‐value	Beta (SE)	*r* ^2^	*P*‐value
SAGE score	−0.036 (0.01)	0.001	**0.0008**	−0.027 (0.01)	0.0005	**0.009**	−0.029 (0.009)	0.0007	**0.002**	−0.011 (0.01)	4 **×** 10^−6^	0.28
SAGE score + covariates	−0.024 (0.01)	0.0004	0.027	0.001 (0.01)	0	0.92	−0.012 (0.009)	3.8 **×** 10^−5^	0.24	0.0007 (0.01)	0	0.94
Yale‐Penn score	−0.044 (0.01)	0.002	**5 × 10^−5^**	−0.034 (0.01)	0.0009	**0.001**	−0.019 (0.009)	0.0003	0.04	−0.015 (0.01)	9.4 **×** 10^−5^	0.15
Yale‐Penn score + covariates	−0.048 (0.01)	0.002	**1 × 10^−5^**	−0.017 (0.01)	0.0002	0.09	−0.004 (0.009)	0	0.66	−0.012 (0.01)	3 × 10^−5^	0.25
Meta‐analysis score	−0.046 (0.01)	0.002	**3.6 × 10^−5^**	−0.035 (0.01)	0.001	**0.0009**	−0.028 (0.009)	0.0009	**0.003**	−0.015 (0.01)	0.0001	0.152
Meta‐analysis score + covariates	−0.034 (0.01)	0.0009	**0.002**	−0.009 (0.01)	0	0.36	−0.01 (0.009)	7.2 × 10^−6^	0.31	−0.005 (0.01)	0	0.6

Results are shown correcting for age + sex + four MDS components in the first instance, and with the addition of social deprivation (SIMD) education in the full model (score + covariates). *P*‐values highlighted in bold remain significant after FDR correction for multiple comparisons. Polygenic risk scores derived from SNPs with a GWAS *P*‐value ≤ 0.5 are presented.

### Relationship of polygenic risk for alcohol dependence to cognitive function

A significant negative association between polygenic risk for alcohol dependence and performance on the verbal fluency test was observed (SAGE: β = −0.036, *P* = 0.0008; Yale‐Penn: β = −0.044, *P* = 0.00005) (Table 2). A significant negative association between Mill Hill Vocabulary and alcohol dependence polygenic risk was also found (SAGE: β = −0.027, *P* = 0.009; Yale‐Penn: β = −0.034, *P* = 0.001) although the SAGE *P*‐value did not withstand correction for multiple testing. Digit symbol coding was negatively associated with polygenic risk for alcohol dependence (SAGE: β = −0.029, *P* = 0.002; Yale‐Penn: β = −0.019, *P* = 0.04); however, the replication in the Yale‐Penn dataset is not significant after correction for multiple testing. All associations between PGRS and cognitive measures have been corrected for self‐reported alcohol consumption.

Education is strongly correlated with performance on the Mill Hill Vocabulary test and the number of years spent in education (Supporting Information Table S2) and SIMD and intelligence are genetically correlated in this sample (Marioni *et al*. 2014). When education and SIMD were added as covariates to the model, the association between alcohol dependence risk score and cognitive function was substantially attenuated and no longer significant for Mill Hill Vocabulary and digit symbol coding. Polygenic risk for alcohol dependence was significantly associated with verbal fluency and the effect sizes remained similar or reduced by ∼33 percent after controlling for education and SIMD (SAGE: β = −0.024, *P* = 0.027; Yale‐Penn: β = −0.048, *P* = 0.00001) (Table 1), although the SAGE score association did not withstand correction for multiple testing.

Previous epidemiological studies have found a positive association between alcohol consumption and cognitive ability (Britton, Singh‐Manoux & Marmot 2004; Corley *et al*. 2011) and this was replicated in GS:SFHS (Supporting Information Table S2). The proportion of variance in cognitive test scores explained by PGRS is shown in Fig. 1 (less than 0.2 percent in all cases), where only measures of cognitive ability that are significantly associated with both SAGE and Yale‐Penn PGRS are presented.

**Figure 2:**
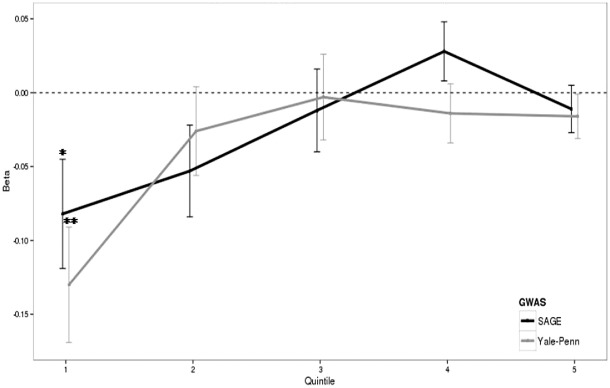
Relationship of alcohol polygenic risk score to MHV in SIMD quintiles

An interaction between SIMD and polygenic risk was observed when Mill Hill Vocabulary was analysed (SAGE: β = 0.026, *P* = 0.01; Yale‐Penn: β = 0.024, *P* = 0.02) (Table 3) but this did not withstand correction for multiple testing. When the meta‐analysed score was tested a significant interaction was also found (β = 0.031, *P* = 0.004), and this remained significant after correction for multiple testing. For illustrative purposes, Fig. 2 shows the association between PGRS and Mill Hill Vocabulary in each quintile of SIMD rank. The impact of alcohol dependence polygenic risk on Mill Hill Vocabulary scores appears most pronounced in individuals in the most socially deprived quintile (1), whereas in the least socially deprived quintile (5), polygenic risk has almost no effect on Mill Hill Vocabulary performance (Fig. 2).

**Table 3:** Association of the interaction term SIMD*polygenic risk score with cognitive variables previously shown to be associated with polygenic risk for alcohol dependence

Polygenic risk score	Verbal fluency		Mill Hill vocabulary		Digit symbol coding	
	Beta (SE)	*P*‐value	Beta (SE)	*P*‐value	Beta (SE)	*P*‐value
SAGE score * SIMD	−0.0003 (0.01)	0.98	0.026 (0.01)	0.01	0.019 (0.009)	0.04
Yale‐Penn * SIMD	−0.0035 (0.01)	0.75	0.024 (0.01)	0.02	0.016 (0.009)	0.09
Meta‐analysis * SIMD	−0.0018 (0.01)	0.87	0.031 (0.01)	**0.004**	0.023 (0.009)	0.01

Results shown are corrected for age, sex, four MDS components; and SIMD and polygenic risk score are included as main effects. Polygenic risk scores derived from SNPs with a GWAS *P*‐value ≤ 0.5 are presented. Bold highlighted *P*‐value is significant after FDR correction.

## Discussion

We find that polygenic risk for alcohol dependence is positively correlated with alcohol consumption in this Scottish population‐based sample using scores derived from two independent GWAS of alcohol dependence. Individuals with a higher genetic load for alcohol dependence reported consuming significantly more alcohol. Despite alcohol consumption being positively correlated with cognitive ability in this sample, alcohol dependence polygenic risk is negatively associated with three measures of cognitive function: digit symbol coding, Mill Hill Vocabulary and verbal fluency. These associations were independent of self‐reported alcohol consumption. When education and social deprivation were added as covariates to the models, only the negative association with verbal fluency remained. These data suggest that lower cognitive functioning may precede alcohol dependence in individuals with a high genetic loading for the disorder, particularly in the domain of executive function (verbal fluency). Alcohol dependence polygenic risk is negatively correlated with SIMD and education. Individuals who carry more alcohol dependence risk alleles tend to live in regions of social deprivation and have spent fewer years in education. This may contribute to the increased prevalence of harmful drinking and alcohol misuse by individuals living in regions of social deprivation. The amount of variance explained by polygenic risk for alcohol dependence across all traits was less than 0.3 percent.

Other studies have found that alcohol consumption is positively correlated with socio‐economic status (Corley *et al*. 2011; Grittner *et al*. 2012) and a study of young adults in Britain found a positive relationship between education level and alcohol consumption (Huerta & Borgonovi 2010). However, despite higher socio‐economic classes consuming more alcohol, individuals living in areas of social deprivation carry the burden of problem drinking and alcohol‐related disease (Bromley *et al*. 2012). The Scottish Health Survey 2012 found that men in low‐income households were most likely to engage in harmful drinking behaviour. Furthermore, a recent study found that men living in Scotland's most deprived area (SIMD quintile 1) were significantly more likely to have an alcohol use disorder than men living in the least deprived area (SIMD quintile 5) (32 percent versus 21 percent) (Bromley *et al*. 2012). In the Welsh Health Survey (2003/2004–2007), respondents in the most socially deprived regions reported the most binge drinking (Fone *et al*. 2013). In the present study, we find that alcohol consumption is positively correlated with socio‐economic status but that individuals living in areas of social deprivation in Scotland tend to carry more alcohol dependence risk alleles. This may be one reason that individuals in areas of social deprivation are more likely to develop alcohol use disorders despite consuming less alcohol overall than the rest of the population. A previous study of GS:SFHS found social deprivation to have a significant genetic component (Marioni *et al*. 2014) and we find that this overlaps with common genetic variation that increases risk for alcohol dependence. While it is clear that social deprivation is a potent environmental risk factor for alcohol dependence, future studies should consider that there may be a genetic component to these exposures that is relevant for alcohol dependence.

Cognitive deficits are common in alcohol‐dependent individuals, particularly in the domain of executive function (Fernandez‐Serrano *et al*. 2010). However, a recent meta‐analysis of cognitive deficits in alcoholism found that most cognitive impairments abate a year after alcohol detoxification (Stavro, Pelletier & Potvin 2013). We find that polygenic risk for alcohol dependence is associated with poorer cognitive ability in some domains, particularly on verbal fluency—a measure of executive function, independent of alcohol consumption. Our findings are supported by family studies showing that non‐dependent offspring of alcoholics score lower on tests of executive function and language than those with a negative family history of alcoholism (Tapert & Brown 2000; Nigg *et al*. 2004; Gierski *et al*. 2013). Furthermore, higher childhood IQ is associated with fewer alcohol‐induced hangovers in adulthood (Batty *et al*. 2006). It is possible that lower cognitive ability is a risk factor for alcohol dependence in individuals with a high genetic risk for the disorder.

In our study, the strength of the negative relationship between alcohol dependence polygenic risk and digit symbol coding and Mill Hill Vocabulary scores was attenuated when social deprivation and education were added as covariates. This may be because education and social deprivation are strongly correlated with Mill Hill Vocabulary, and much of the variance in Mill Hill Vocabulary is removed when adjusting for these variables. The causal direction is moot: higher verbal ability might be the result of more education and living in a more affluent area, or vice versa, or influences might flow dynamically in both directions (Deary & Johnson 2010). It is important to recognize that when education is added as a control variable it is not just as an environmental factor, partly genetically influenced cognitive ability also drives educational attainment to some degree.

We find no effect of social deprivation or education on the association between verbal fluency and polygenic risk for alcohol dependence. Verbal fluency is believed to be a frontal lobe process (Fuster 2008) as patients with frontal lobe damage are significantly impaired on tests of phonemic word fluency (Robinson *et al*. 2012). Damage to the frontal lobes induces behaviour typically associated with addiction, such as the inability to defer immediate rewards for greater delayed rewards (Berlin, Rolls & Kischka 2004). Indeed, chronic alcohol consumption is associated with metabolic and morphological changes in the frontal lobes, particularly the prefrontal cortex (Adams *et al*. 1993; Pfefferbaum *et al*. 1997). Grey matter reductions in the prefrontal cortex of alcoholics are correlated with worse executive function (Chanraud *et al*. 2007). Here we show that polygenic risk for alcohol dependence is negatively associated with phonemic verbal fluency, suggesting that frontal lobe deficits may be pre‐existing risk factors for the development of alcohol dependence. However, because the present study is correlational in nature, evaluation of this relationship requires a prospective design in which cognitive function is evaluated prior to the onset of alcohol dependence.

A recent study of healthy young adults found that exposure to social and personal adversity is a risk factor for executive function deficits in individuals with a positive family history of alcohol dependence (Lovallo *et al*. 2013). In support of this, we find the impact of polygenic risk for alcohol dependence on Mill Hill Vocabulary to be greatest in individuals living in the most socially deprived areas. However, we also find that individuals living in social deprivation tend to carry more alcohol dependence risk alleles, which in turn is correlated with worse performance on Mill Hill Vocabulary tests. Therefore, a gene × environment interaction cannot be readily differentiated from a gene–environment correlation. Furthermore, the interaction between PGRS and social deprivation only withstood correction for multiple testing when the meta‐analysed risk score was used and therefore these results need to be interpreted cautiously until replicated in an independent sample.

There are some limitations to this study. Information on alcohol dependence was not available in the GS:SFHS dataset and therefore we do not know to what extent subjects with alcohol dependence are influencing our analyses. In an independent study, the Scottish Health Survey 2012 found that 21 percent of men and 11 percent of women were classed as hazardous drinkers. However, as we are able to control for alcohol consumption it is unlikely to be a significant confounder, although our measure of alcohol consumption is based on self‐report and potentially underestimated. Another limitation is that the number of individuals in the original GWAS for alcohol dependence was relatively low (SAGE *n* = 2377, Yale‐Penn *n* = 2750). Previous studies using PGRS have used GWAS datasets comprising 7000–22 000 individuals (Purcell *et al*. 2009; McIntosh *et al*. 2013). However, we were able to find a robust association with alcohol consumption using our risk scores suggesting they are valid tools to investigate the genetic overlap between disorders. The variation in weekly alcohol consumption explained by the alcohol dependence PGRS is low, less than 0.1 percent. We created a meta‐analysed PGRS that utilized summary statistics from the Yale‐Penn and SAGE GWAS combined (*n* = 5127) to try to increase the amount of variance explained in our traits of interest. This score explained 0.3 percent of the variance in alcohol consumption. A large GWAS of schizophrenia (*N* ∼ 150 000) was used to create PGRS in an independent schizophrenia cohort. These scores explained 7 percent of the variance in schizophrenia (Schizophrenia Working Group of Psychiatric Genetics Consortium 2014) and demonstrate that as the size of a discovery GWAS increases the amount of variance explained by PGRS increases. Considering that the genetic overlap between alcohol consumption and abuse is not perfect (rG = 0.61) (Dick *et al*. 2011) and the original GWAS for alcohol dependence had fewer individuals, it is understandable that the variance explained by PGRS is low. Finally, not all of the associations we report withstand correction for multiple testing. The associations between digit symbol coding, education and alcohol dependence PGRS were modest and do not survive correction for multiple testing across both datasets. However, it is notable that Mill Hill Vocabulary, digit symbol coding and education are nominally associated with alcohol dependence polygenic risk with the same direction of effect observed for each score.

The data presented in this study provide evidence that polygenic risk for alcohol dependence associates with alcohol consumption, social deprivation and some domains of cognitive ability in a large population‐based sample. These findings allow us to understand better the biological mechanisms underlying these traits and their associations. Cognitive ability may not only be a result of chronic alcohol consumption, but a pre‐disposing risk factor for the development of alcohol dependence, although longitudinal data are required to test this hypothesis. By understanding the relationship among alcohol dependence, social deprivation and cognitive ability, we may identify individuals at high risk to develop alcohol dependence and inform health interventions to reduce the burden of alcohol misuse on society. Thus, prospective evaluation of the findings reported here may create a basis for focused prevention efforts.

## Disclosure/Conflict of Interest

A.M.M. has received financial support from Pfizer (formerly Wyeth), Janssen and Lilly. A.M.M. has done consultancy work for Roche Pharmaceuticals. H.R.K. has been a consultant or advisory board member with Alkermes, Lilly, Lundbeck, Otsuka, Pfizer and Roche. He is a member of the Alcohol Clinical Trials Initiative (ACTIVE) of the American Society of Clinical Psychopharmacology, which is supported by AbbVie, Ethypharm, Lilly, Lundbeck and Pfizer and has a US patent pending, entitled ‘Test for Predicting Response to Topiramate and Use of Topiramate’. L.J.H. has received financial support from Pfizer for work unrelated to the present study.

## Authors Contribution

TKC and AMM had full access to all of the data in the study and take responsibility for the integrity of the data and the accuracy of the data analysis. Drafting of the manuscript: TKC. Critical revision of the manuscript for important intellectual content: All authors. GS:SFHS study concept and design: BHS, SP, LJH, DJP, IJD, AMM, DJM, PAT, CH. Yale‐Penn and SAGE study concept, design and analysis : AHS, JG, HRK, LAF. Statistical analysis: TKC. Administrative, technical or material support: CH, AMFP, DJM, AHS and LSH. Acquisition, analysis or interpretation of data: All authors. Study supervision: AMM, IJD, DJP.

## Funding

The Chief Scientist Office of the Scottish Government and the Scottish Funding Council provided core support for Generation Scotland. GS:SFHS was funded by a grant from the Scottish Government Health Department, Chief Scientist Office (No. CZD/16/6). Genotyping services for a part of the Yale GWAS study were provided by the Center for Inherited Disease Research (CIDR) and Yale University (Center for Genome Analysis). CIDR is fully funded through a federal contract from the National Institutes of Health to The Johns Hopkins University (Contract No. N01‐HG‐65403). The publicly available datasets used for the analyses described in this manuscript were obtained from dbGaP at http://www.ncbi.nlm.nih.gov/projects/gap/cgi‐bin/study.cgi?study_id=phs000092.v1.p1 through dbGaP accession number phs000092.v1.p. Funding support for the Study of Addiction: Genetics and Environment (SAGE) was provided through the NIH Genes, Environment and Health Initiative [GEI] (U01 HG004422). SAGE is one of the genome‐wide association studies funded as part of the Gene Environment Association Studies (GENEVA) under GEI. Assistance with phenotype harmonization and genotype cleaning, as well as with general study coordination, was provided by the GENEVA Coordinating Center (U01 HG004446). Assistance with data cleaning was provided by the National Center for Biotechnology Information. Support for collection of datasets and samples was provided by the Collaborative Study on the Genetics of Alcoholism (COGA; U10 AA008401), the Collaborative Genetic Study of Nicotine Dependence (COGEND; P01 CA089392) and the Family Study of Cocaine Dependence (FSCD; R01 DA013423). Funding support for genotyping, which was performed at the Johns Hopkins University Center for Inherited Disease Research, was provided by the NIH GEI (U01HG004438), the National Institute on Alcohol Abuse and Alcoholism, the National Institute on Drug Abuse, and the NIH contract ‘High throughput genotyping for studying the genetic contributions to human disease’ (HHSN268200782096C). The authors TKC and AMM acknowledge with gratitude the financial support received for this work from the Dr Mortimer and Theresa Sackler Foundation. PAT, DJP, IJD and AMM are members of The University of Edinburgh Centre for Cognitive Ageing and Cognitive Epidemiology, part of the cross council Lifelong Health and Wellbeing Initiative (MR/K026992/1). Funding from the Biotechnology and Biological Sciences Research Council (BBSRC) and Medical Research Council (MRC) is gratefully acknowledged, supported in part by National Institutes of Health grants RC2 DA028909, R01 DA12690, R01 DA12849, R01 DA18432, R01 AA11330, R01 AA017535, P50 AA12870, MSTP T32GM07205 and CTSA 8UL1TR000142. This work is supported by the Wellcome Trust through a Strategic Award, reference 104036/Z/14/Z.

## Supporting information


**Figure S1** MDS plot showing first and second MDS component distinguishing GS:SFHS genotyped individuals from 11 other ethnic populations in HapMap. (CEU = Utah Residents with Northern and Western European Ancestry, CHB = Han Chinese in Beijing, YRI = Yoruba in Ibadan, TSI = Toscans in Italy, JPT = Japanese in Tokyo, CHD = Chinese in Denver, MEX = Mexican ancestry in Los Angeles California, GIH = Gujarati Indians in Houston, ASW = African Ancestry in South West USA, LWK = Luhya in Wubuye, MKK = Maasai in Kinyawa)
**Table S1** Relationship between polygenic risk score with chromosome 4 SNPs excluded and alcohol consumption. Results are shown correcting for age + sex + 4 MDS components and for education and SIMD when covariates are added. Polygenic risk scores derived from SNPs with a GWAS *P*‐value ≤ 0.5 are presented
**Table S2** Pearson's correlations between alcohol consumption, cognitive variables, SIMD and education in unrelated members of the GS:SFHS cohort (*n* = 6413). MHV = Mill Hill Vocabulary, VF = Verbal Fluency, DSC = Digit Symbol Coding, LM = Logical Memory, SIMD = Scottish Index of Multiple Deprivation. All correlations significant at *P* ≤ 1.46 **×** 10^−6^
