# Supplementary material for: Polygenic risk for alcohol dependence associates with alcohol consumption, cognitive function and social deprivation in a population‐based cohort
Source: Addict Biol. 2015 Apr 10;21(2):469–80. doi: 10.1111/adb.12245 (PMC4600406; doi:10.1111/adb.12245)
Supplement: Supplementary file 1 — Figure S1 MDS plot showing first and second MDS component distinguishing GS:SFHS genotyped individuals from 11 other ethnic populations in HapMap. (CEU = Utah Residents with Northern and Western European Ancestry, CHB = Han Chinese in Beijing, YRI = Yoruba in Ibadan, TSI = Toscans in Italy, JPT = Japanese in Tokyo, CHD = Chinese in Denver, MEX = Mexican ancestry in Los Angeles California, GIH = Gujarati Indians in Houston, ASW = African Ancestry in South West USA, LWK = Luhya in Wubuye, MKK = Maasai in Kinyawa) Table S1 Relationship between polygenic risk score with chromosome 4 SNPs excluded and alcohol consumption. Results are shown correcting for age + sex + 4 MDS components and for education and SIMD when covariates are added. Polygenic risk scores derived from SNPs with a GWAS P‐value ≤ 0.5 are presented Table S2 Pearson's correlations between alcohol consumption, cognitive variables, SIMD and education in unrelated members of the GS:SFHS cohort (n = 6413). MHV = Mill Hill Vocabulary, VF = Verbal Fluency, DSC = Digit Symbol Coding, LM = Logical Memory, SIMD = Scottish Index of Multiple Deprivation. All correlations significant at P ≤ 1.46 × 10^−6^ [file ADB-21-469-s001.docx]

Supplemental Tables

|  | **SAGE polygenic risk score** | | **Yale-Penn polygenic risk score** | |
| --- | --- | --- | --- | --- |
| **Trait** | **Statistics** | **P-value** | **Statistics** | **P-value** |
| Alcohol | β=0.034, r^2^=0.001 | 0.001 | β=0.036, r^2^=0.001 | 0.0006 |
| Alcohol + covariate adjustment | β=0.04, r^2^=0.002 | 0.00008 | β=0.038, r^2^=0.001 | 0.0005 |

eTable 1) Relationship between polygenic risk score with chromosome 4 SNPs excluded and alcohol consumption. Results are shown correcting for age + sex + 4 MDS components and for education and SIMD when covariates are added. Polygenic risk scores derived from SNPs with a GWAS p-value ≤ 0.5 are presented.

|  | **Alcohol consumption** | **MHV** | **VF** | **DSC** | **LM** | **SIMD** | **Education** |
| --- | --- | --- | --- | --- | --- | --- | --- |
| **Alcohol consumption** | 1 | - | - | - | - | - | - |
| **MHV** | 0.11 | 1 | - | - | - | - | - |
| **VF** | 0.11 | 0.39 | 1 | - | - | - | - |
| **DSC** | 0.05 | 0.10 | 0.27 | 1 | - | - | - |
| **LM** | 0.05 | 0.26 | 0.16 | 0.28 | 1 | - | - |
| **SIMD** | 0.07 | 0.23 | 0.13 | 0.14 | 0.10 | 1 | - |
| **Education** | 0.09 | 0.34 | 0.23 | 0.31 | 0.23 | 0.21 | 1 |

eTable 2) Pearson’s correlations between alcohol consumption, cognitive variables, SIMD and education in unrelated members of the GS:SFHS cohort (N=6413). MHV = Mill Hill Vocabulary, VF= Verbal Fluency, DSC=Digit Symbol Coding, LM=Logical Memory, SIMD= Scottish Index of Multiple Deprivation. All correlations significant at p ≤ 1.46 x 10^-6^.


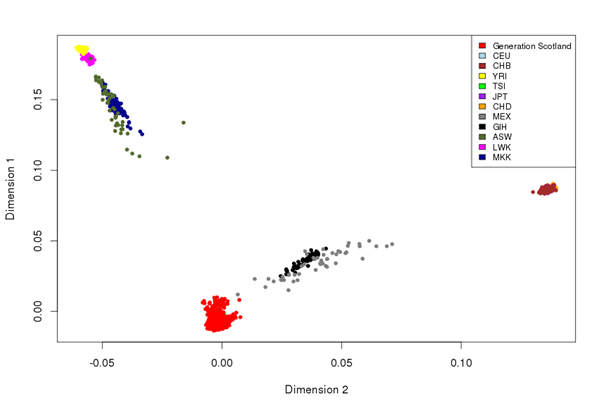


eFigure 1) MDS plot showing first and second MDS component distinguishing GS:SFHS genotyped individuals from 11 other ethnic populations in HapMap. (CEU= Utah Residents with Northern and Western European Ancestry, CHB = Han Chinese in Beijing, YRI = Yoruba in Ibadan, TSI = Toscans in Italy, JPT = Japanese in Tokyo, CHD = Chinese in Denver, MEX = Mexican ancestry in Los Angeles California, GIH = Gujarati Indians in Houston, ASW= African Ancestry in South West USA, LWK=Luhya in Wubuye, MKK=Maasai in Kinyawa)
